# Longitudinal analysis of rhesus macaque metabolome during acute SIV infection reveals disruption in broad metabolite classes

**DOI:** 10.1128/jvi.01634-24

**Published:** 2025-02-06

**Authors:** Andrew Hudson, Peng Wu, Kyle W. Kroll, Brady Hueber, Griffin Woolley, Pixu Shi, R. Keith Reeves

**Affiliations:** 1 Division of Innate and Comparative Immunology, Center for Human Systems Immunology, Duke University School of Medicine 12277 , Durham, North Carolina, USA; 2 Department of Biostatistics and Bioinformatics, Duke University School of Medicine 12277 , Durham, North Carolina, USA; 3 Department of Surgery, Duke University School of Medicine 12277 , Durham, North Carolina, USA; University Hospital Tübingen, Tübingen, Germany

**Keywords:** immunometabolism, HIV, proteomics

## Abstract

Persons living with HIV experience significant metabolic dysregulation, frequently resulting in immune and other cellular dysfunction. However, our understanding of metabolism and its relationship to immunity in the context of HIV remains incompletely understood, especially as it relates to the acute and early chronic phases of HIV infection. Herein, we employed mass spectrometry and a simian immunodeficiency virus (SIV)-infected rhesus macaque model to characterize changes in over 500 plasma metabolites throughout SIV infection. This broad metabolomic approach recapitulated known pathogenic signatures of HIV, such as a perturbed tryptophan/kynurenine ratio, but also identified novel metabolic changes. We observed a general decrease in plasma amino acid concentrations, with the notable exceptions of elevated aspartate and glutamate. Acute infection was marked by a transient increase in lactate dehydrogenase activity, indicating a shift toward anaerobic metabolism. Indoleamine 2,3-dioxygenase activity, defined by the kynurenine/tryptophan ratio, was significantly increased in both acute and chronic phases and strongly correlated with viral load. These results provide a comprehensive characterization of metabolic fluctuations during early lentiviral infection, furthering our understanding of the crucial interplay between metabolism and immune response. Our findings highlight systemic metabolic consequences of infection and provide potential targets for therapeutic intervention or biomarkers of disease progression.

**IMPORTANCE:**

Despite significant advances in antiretroviral therapy and pre-exposure prophylaxis, HIV remains a global challenge. Understanding the underlying immune mechanisms is critical for improving HIV control and therapeutic development. Cellular metabolism represents a crucial yet underappreciated area of immune system function. Metabolite availability and metabolic pathway preferences directly influence the functional response capacity of immune cells and are highly dysregulated during HIV infection. To further the understanding of metabolic impacts of HIV infection, we utilized cutting-edge mass spectrometry-based metabolome interrogation to measure over 500 metabolites using an acute simian immunodeficiency virus infection model in rhesus macaques. Our comprehensive analysis provides insights into the dynamic metabolic landscape throughout early infection, revealing both known and novel metabolic signatures. These findings enhance our understanding of the complex interplay between metabolism and immunity in lentiviral infections, potentially informing new strategies for early detection, prevention, and treatment of HIV.

## INTRODUCTION

Human immunodeficiency virus (HIV) is an ongoing global health burden with approximately 39 million people living with HIV (PLWH), but only 30 million on antiretroviral therapy (ART). Despite the success of ART in suppressing viremia, PLWH continue to face increased comorbidity risks, such as chronic inflammation, accelerated aging, immune activation, and higher incidences of metabolic disorders such as obesity, diabetes mellitus, and nutrient deficiency (1–4). These persistent health challenges underscore the need for a deeper understanding of the complex interplay between HIV and host metabolism.

Metabolism and the immune system are dynamically linked, with inflammation driving metabolic requirements and systemic metabolites in turn influencing immune cell function (5). Amino acids in particular play crucial roles in various immune cell functions. For instance, glutamine is essential for T cell proliferation and B cell production (6, 7), while also driving natural killer (NK) cell function (8). The metabolic state of B cells, particularly their glutamine utilization, directly impacts antibody production and quality (9, 10), which is crucial for mounting an effective immune response against HIV (11). Methionine is critical for T cell activation (12, 13), and arginine is necessary for T cell and macrophage function (14). Moreover, tryptophan metabolism, regulated by indoleamine 2,3-dioxygenase (IDO), affects T cell responses and is often dysregulated in HIV infection (15, 16). These metabolic pathways not only influence cellular immune responses but also impact the overall systemic metabolic profile in PLWH.

HIV infection is associated with various metabolic disorders, even in the era of ART. These include lipodystrophy, characterized by abnormal fat distribution and often accompanied by insulin resistance and dyslipidemia (17–19). HIV-associated wasting syndrome, although less common with modern ART, still affects some patients and involves significant loss of body mass (20). Additionally, PLWH have an increased risk of cardiovascular disease, partly due to metabolic alterations such as dyslipidemia and insulin resistance (21–23). Mitochondrial toxicity, potentially exacerbated by some ART medications, can lead to lactic acidosis and hepatic steatosis (24, 25). Understanding these metabolic complications is crucial for improving long-term outcomes for PLWH and may inform strategies to enhance immune function and reduce comorbidities.

Metabolic processes play a crucial role in modulating the susceptibility of CD4^+^ T cells to HIV infection and the functional capabilities of NK cells. During HIV infection, the virus preferentially targets CD4^+^ T cells exhibiting heightened metabolic activity, characterized by elevated levels of oxidative phosphorylation and glycolysis, irrespective of the cells’ activation state (26, 27). This preference highlights the intricate relationship between cellular metabolism and viral replication. Interestingly, partially inhibiting glycolysis not only hampers HIV infection *in vitro* across all CD4^+^ T cell subsets but also reduces the viability of pre-infected cells and prevents HIV amplification in cells derived from PLWH (28). These findings suggest that metabolic interventions could potentially be leveraged as part of HIV treatment strategies, opening new areas of investigation for therapeutic development.

The metabolic context is fundamental to immune cell activity across various cell types. NK cells, critical for antiviral immunity, increase nutrient uptake and reprogram their metabolic machinery during viral infections (29, 30). In viremic HIV-1 infection, NK cells exhibit impaired oxidative phosphorylation, reduced metabolic reserve, and mitochondrial defects, coinciding with decreased interferon response (29, 30). HIV-specific CD8^+^ T cells from non-controllers of HIV show more dependence on glycolysis and use less diverse metabolic pathways than those of HIV controllers (31), highlighting a role of metabolic plasticity in anti-HIV activity. Similarly, the metabolic state of B cells influences their ability to produce high-affinity antibodies against pathogens such as HIV (32, 33), with alterations in glucose metabolism and fatty acid oxidation affecting antibody class switching and somatic hypermutation (34–36).

Despite this growing body of knowledge, the impact of lentiviral infection on the circulating metabolome remains incompletely characterized, especially during the initial stages of HIV infection prior to the development of immunodeficiency. This timeframe is of critical importance for the development of preventive therapies, vaccine design, and early detection strategies. To address these gaps in knowledge, we employed a rhesus macaque (RM) simian immunodeficiency virus (SIV) model to examine how the circulating metabolome is impacted during very early stages of lentiviral infection. This approach allows us to track metabolic changes longitudinally from pre-infection through acute and early chronic stages, providing insights that may be challenging to obtain in human studies. By elucidating these early metabolic alterations, we aim to identify potential targets for therapeutic intervention and biomarkers for early HIV detection.

## RESULTS AND DISCUSSION

**Fig 1:**
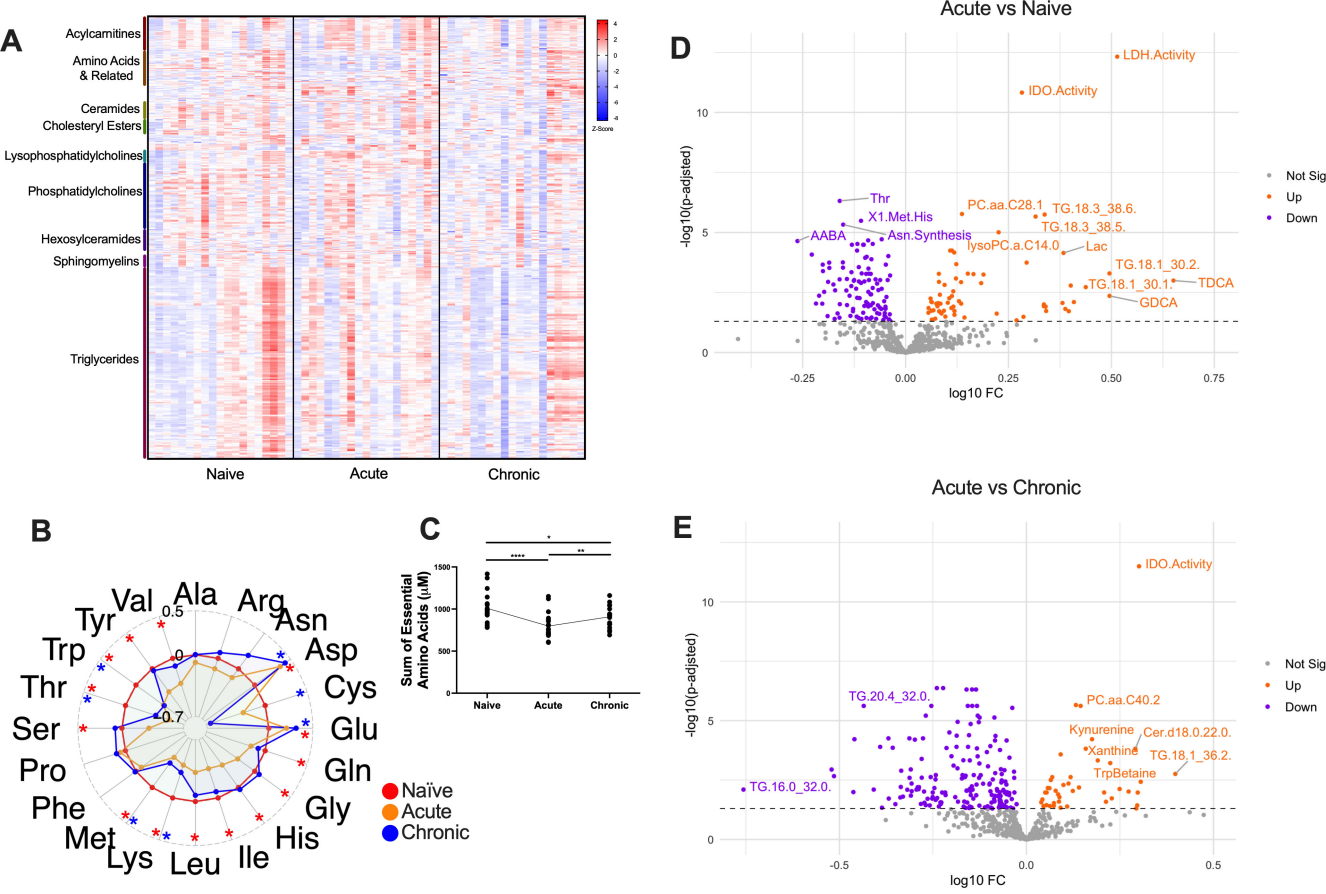
SIV causes broad metabolic perturbations during acute and chronic infection. (**A**) Normalized concentrations of 508 metabolites in rhesus macaque plasma (*n* = 19) across three stages of SIV infection (naïve = day −21 or day 0 post-infection [p.i.]; acute = day 7 p.i.; chronic = day 50 or day 70 p.i.). The largest metabolite families are annotated. (**B**) Radar plot of log2 fold changes of amino acid concentrations for acute and chronic compared to naïve. Colored asterisks indicate significant change (*P* ≤ 0.05) between infected and naïve timepoints for acute-naïve comparison (red asterisks) and chronic-naïve comparison (blue asterisks). (**C**) Sum of concentrations of essential amino acids across timepoints. (**D, E**) Volcano plots depicting fold change of plasma metabolites and metabolism indicators between acute and naïve (**D**) and between chronic and naïve (**E**). For longitudinal metabolite concentrations and metabolism indicators, *P*-values were calculated by paired *t*-test with Benjamini-Hochberg adjustment. * *P* < 0.05; **P < 0.01; ***P < 0.001; ****P < 0.0001.

To characterize specific metabolic changes during early lentiviral infection, we conducted a longitudinal nonhuman primate study using RM infected with SIV. We employed mass spectrometry to assess 508 soluble metabolites encompassing various metabolite classes during infection (Fig. 1A) to provide a comprehensive overview of key metabolic pathways potentially affected by SIV infection. Our analysis reveals broad changes in the metabolic landscape during acute and chronic SIV infection, including alterations in amino acid concentrations, enzyme activities, and lipid profiles. Our acute phase timepoint was immediately prior to peak viral load and matched the subsequent set point viral load maintained into chronic phase (Fig. S1A). Serum glucose concentrations were consistent throughout the study, suggesting a negligible role of dietary variation and environmental factors on general metabolic status (Fig. S1B).

We observed a general decrease of canonical amino acid concentrations in plasma during SIV infection (Fig. 1B). Specifically, glutamine, glycine, histidine, isoleucine, leucine, serine, tyrosine, and valine significantly decreased in the acute phase compared to baseline, while lysine, methionine, threonine, and tryptophan also decreased in the acute phase and additionally failed to recover by the start of the chronic phase (Fig. 1B). The sum of essential amino acid concentrations was also consistently diminished post-infection (p.i.) (Fig. 1C). These findings align with previously reported associations of HIV with amino acid depletion, and decreased arginine, glycine, methionine, serine, tryptophan, and tyrosine in PLWH have been shown to correlate with inflammation and insulin resistance (1, 37).

On the immunometabolic scale, amino acid availability is critical for lymphocyte function. Methionine plays a crucial role in T cell activation, and free glutamine exerts multifaceted effects across several cell types: glutamine feeds glutaminolysis for T cell proliferation and B cell antibody production, and drives NK cell function through the mTORC1-cMYC signaling axis (38). Both methionine and glutamine were significantly depleted from baseline in the acute timepoint, and methionine remained diminished throughout the study (Fig. 1B). This sustained depletion could have long-term consequences for T cell function and overall immune response. Additionally, when comparing acute to pre-infection timepoints, significant upregulation of IDO and lactate dehydrogenase (LDH) activity and triglycerides were observed (Fig. 1D). A small subset of specific phosphatidylcholines (PCs) showed significant increases during acute infection, but this was not representative of phosphatidylcholines as a whole. In fact, the overall PC:choline ratio decreased in the acute phase. These nuanced changes in phosphatidylcholine levels are noteworthy, as these lipids play crucial roles in cell membrane structure and signaling (39, 40). The complex remodeling of phospholipid metabolism during early SIV infection, including both increases in specific phosphatidylcholines and an overall decrease in the PC:choline ratio, may reflect targeted alterations in membrane composition or cellular stress responses. However, the exact biological significance of these changes in the context of HIV/SIV infection requires further investigation.

**Fig 2:**
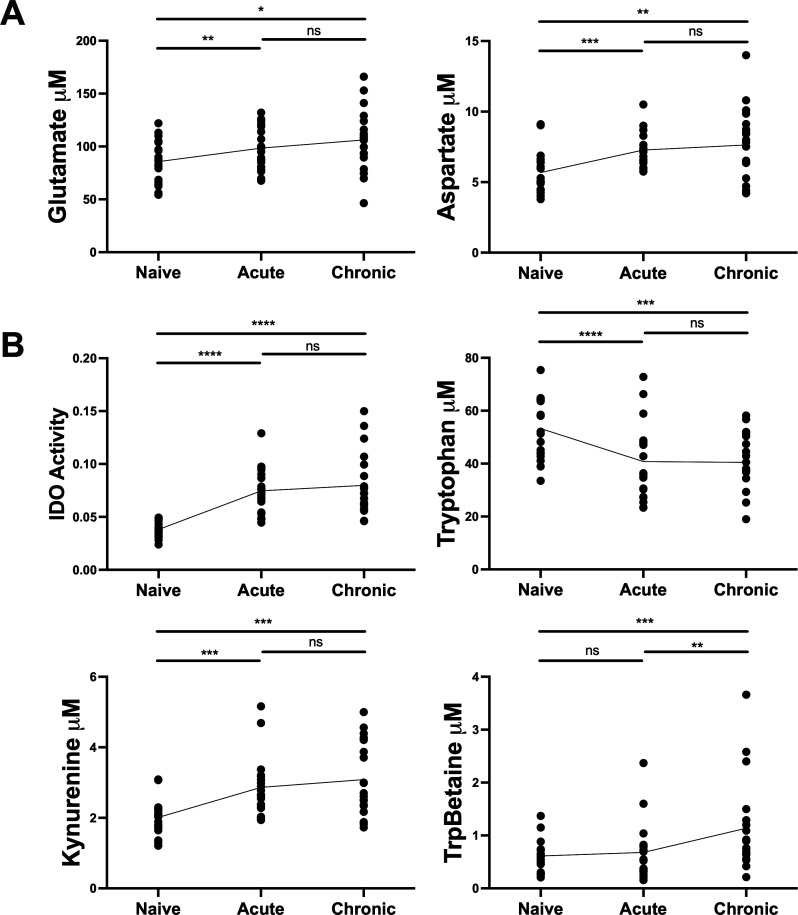
SIV infection drives robust activation of the IDO pathway. (**A**) Longitudinal concentrations of glutamate and aspartate during infection. (**B**) IDO activity (kynurenine/tryptophan) and concentrations of tryptophan, kynurenine, and tryptophan betaine (TrpBetaine) across timepoints. *P*-values were calculated by paired *t*-test with Benjamini-Hochberg adjustment. * *P* < 0.05; **P < 0.01; ***P < 0.001; ****P < 0.0001.

When comparing chronic to pre-infection timepoints, we observed sustained upregulation of IDO activity and began to see more amino acid-derived metabolites showing dysregulation, including kynurenine and tryptophan betaine (Fig. 1E). Interestingly, glutamate and aspartate were the only canonical amino acids which increased over the course of infection (Fig. 2A). Elevated plasma glutamate concentrations have been previously demonstrated in PLWH (37), and glutamate has been further shown to inhibit cytotoxic T cell function *in vitro* (41). The observed increase in aspartate, which is closely related to glutamate (42), represents a novel finding in the context of lentiviral infection which could open new avenues for investigating the role of aspartate in SIV/HIV pathogenesis and its potential as a biomarker or therapeutic target. These findings strengthen the hypothesis that lentiviral infection causes sustained metabolic modifications that may be aiding in how these viruses escape immune control. The altered metabolic environment could potentially create conditions favorable for viral persistence and replication while simultaneously impairing effective immune responses.

Further analysis revealed dynamic changes in specific metabolic indicators throughout infection. LDH activity, defined by increased lactic acid and decreased hexose sugar, was significantly upregulated in the acute phase compared to pre-infection (Fig. 1D), with this feature driven by changes in both lactic acid and hexose (Fig. S1C). LDH is a key enzyme in anaerobic metabolism, converting pyruvate into lactate to generate NADP^+^ and thereby diverting resources from oxidative phosphorylation into glycolysis (43). High-serum LDH is linked to T cell depletion and opportunistic infection in PLWH (44), and also may indicate enhanced glycolytic flux and hypoxia, hallmarks of inflammation associated with CD4^+^ T cell infectivity and HIV latency, respectively (28, 45). However, we observed elevated LDH activity strictly during acute infection in this model.

IDO activity was significantly increased in both acute and chronic phases compared to the naïve state (Fig. 1D and E; Fig. 2B). Moreover, IDO activity was the feature most strongly correlated with viral load (Fig. S1D), reinforcing its established role in the immunosuppression of HIV, as IDO is the rate-limiting enzyme in the conversion of tryptophan to kynurenine, a known inhibitor of lymphocyte function (5). HIV-induced perturbations in the tryptophan/kynurenine ratio have been shown to diminish the population of IL-17-secreting helper T cells (T_H_17) and type 3 innate lymphoid cells (ILC3s) in the gastrointestinal mucosa (16, 46–49). Both T _H_17 and ILC3s play crucial roles in mediating host-microbiome interactions and protecting against translocation of inflammatory compounds produced by intestinal flora (16, 50). While the impact of HIV/SIV on IDO activity and its subsequent effects on T_H_17 and ILC3s have been extensively reported in previous studies (51–54), the extent to which these mechanisms are conserved between HIV and SIV models may require addition investigation. In our study, SIV infection not only diminished tryptophan and increased kynurenine concentrations (Fig. 2B), but also increased the abundance of tryptophan betaine (Fig. 2B), another anti-inflammatory tryptophan derivative associated with immune dysregulation in the context of HIV (55).

**Fig 3:**
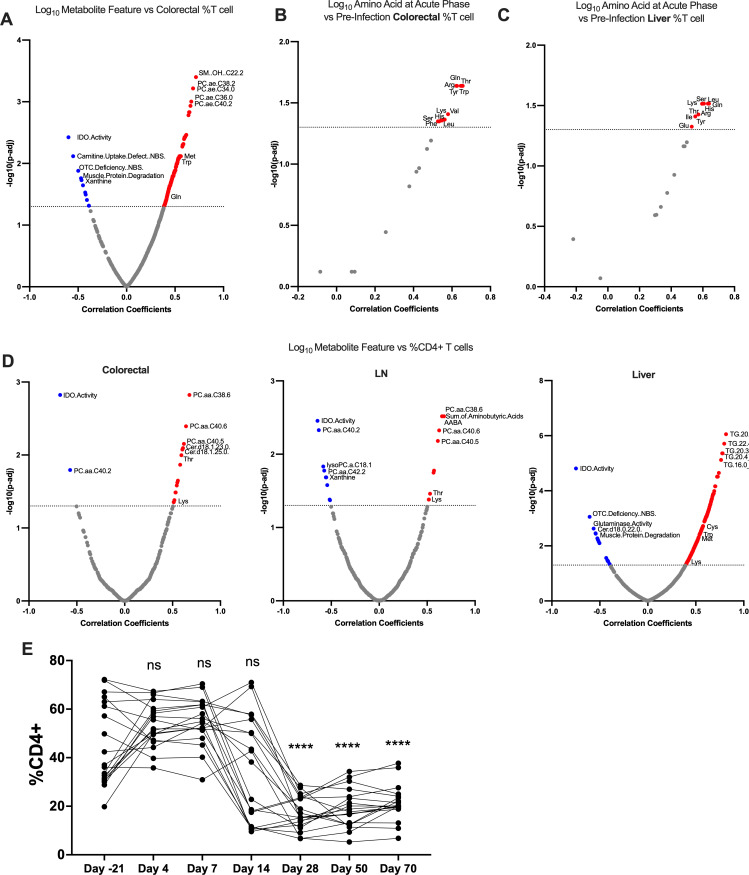
Changes in IDO activity and amino acids caused by SIV infection correspond to shifts in tissue resident T cell populations. (**A**) Correlation of plasma metabolites and T cell frequency of live CD45^+^ cells in colorectal biopsies. (**B, C**) Pre-infection T cell abundance in gut as predictor of acute phase plasma amino acid concentration. Correlation of plasma amino acids at day 7 with pre-infection T cell frequency of live CD45^+^ cells in (**B**) colorectal and (**C**) liver biopsies. (**D**) Correlation of plasma metabolites and CD4^+^ T cell frequency in colorectal, lymph node, and liver biopsies. (**E**) CD4^+^ T cell frequency in periphery over time. One-way analysis of variance was conducted with multiple comparisons against day −21. For volcano plots correlating metabolites with cell populations, unless stated otherwise, comparisons are conducted using data from naïve and chronic timepoints. Blue = negatively correlated metabolite, gray = no significance, red = positively correlated metabolite; dotted line at *P* = −log_10_(0.05). Pearson correlation was conducted with Benjamini-Hochberg adjustment for multiple comparisons. * *P* < 0.05; **P < 0.01; ***P < 0.001; ****P < 0.0001.

IDO activity further correlated with several features of T cell composition in tissues. The frequency of T cells in the gut negatively correlated with plasma IDO activity and positively correlated with several amino acids (Fig. 3A), and pre-infection T cell frequency in the gut and liver was associated with increased plasma amino acid abundance during the acute phase (Fig. 3B and C), suggesting a subtle and thus far uncharacterized protective effect of gut- and liver-resident T cells against the metabolic toll of acute SIV. IDO activity negatively correlated with T_H_ cell frequency in the gut, liver, and lymph nodes (Fig. 3D), potentially owing to the anticipated downtrend of CD4^+^ T cells over the course of infection (Fig. 3E). Our data agree with previous findings that IDO activity and T_H_ cell programmed cell death protein 1 (PD1) expression do not correlate in the periphery in the context of HIV (Fig. S2A); however, we have found that the frequency of PD1^+^ cells among colorectal, lymph node, and liver central memory T_H_ cells positively correlated with plasma IDO (Fig. S2A) (56). PD1 expression of infected cells has been linked to high proviral DNA and resistance to reactivation, and therefore, a portion of these PD1^+^ memory T_H_ cells likely represents a latently infected population increasing over time (Fig. S2B) (57, 58).

While our data generally agree with literature on the metabolic effects of HIV, it is important to acknowledge differences between the RM model and human metabolism. For instance, it has been reported that RMs exhibit greater rates of glutaminolysis than humans, as indicated by high glutamate and low glutamine within erythrocytes and the extracellular compartment (59), though studies diverge on whether glutamine adheres to this trend (60). Furthermore, untreated HIV has been previously shown to increase plasma arginine, a feature we did not observe, possibly owing to substantial differences in baseline arginine concentrations between humans and RM (59, 61). SIV-induced changes in circulating amino acids should be considered in light of interspecific metabolic differences.

In conclusion, using an RM SIV model for early lentiviral infection, our study not only recapitulated known pathogenic signatures of HIV, but also identified novel metabolic changes during early SIV infection. SIV infection generally diminished amino acid levels apart from the closely related aspartate and glutamate, of which the latter has been previously shown to confer immune-exhaustive effects (41). Evidence for IDO activity and its relationship to gut, lymph node, and liver T cell populations further supports an immunosuppressive metabolic milieu throughout early chronic infection. Altogether, our findings not only provide further support to non-human primate models of HIV but also highlight potential new targets for therapeutic intervention. The comprehensive characterization of the evolving metabolic landscape throughout the acute to early chronic SIV time course emphasizes the systemic and immunometabolic consequences of lentiviral infection. These results lay the foundation for examinations of the metabolome in other sites during HIV/SIV infection, such as B cell follicles and other viral reservoirs. Future studies should focus on validating these findings in human HIV infections and exploring how these metabolic changes could be targeted to improve HIV treatment and potentially contribute to cure strategies.

## MATERIALS AND METHODS

### Animal model and experimental design

Nineteen RM were intrarectally infected with SIVmac251 at a dose of 500,000 IU/mL. All animals were housed, and all experimental procedures performed, at a Biomere facility accredited by the Association for the Assessment and Accreditation of Laboratory Animal Care International and registered with the United States Department of Agriculture to conduct research in laboratory animals. Animals were exposed to a 12 hour light/12 hour dark cycle (64°F to 84°F, 30 to 70% room humidity) and were provided filtered tap water *ad libitum* via an automatic watering system as well as Monkey Diet 5038, a diet approved by the Biomere Institutional Animal Care and Use Committee and designed to provide nutrition and enrichment. Telazol (5 to 8 mg/kg, intramuscular; Patterson Veterinary Supply, Devens, MA) was administered to assist in blood collections, mucosal washes, mucosal and liver biopsies, and test article administration. Blood samples were collected using venipuncture. Animals were euthanized with an overdose of pentobarbital, followed by necropsy with tissue collection.

### Sample collection and metabolic analysis

Blood samples taken at naïve timepoints (day −21 or day 0 p.i.), an acute timepoint (day 7 p.i.), and chronic timepoints (day 50 or 70 p.i.). Plasma was isolated from these blood samples for metabolic analysis. Metabolic profiling was performed using the MxP Quant 500 Kit (Biocrates Innsbruck, Austria) in strict accordance with the provided detailed protocol. This specialized kit enabled the assessment of 508 soluble metabolites across various classes by mass spectrometry. Specific metabolic indicators were calculated as follows: LDH activity was defined as the plasma concentration of lactic acid divided by the plasma concentration of hexose; and IDO activity was defined as the plasma concentration of kynurenine divided by the plasma concentration of tryptophan.

### Targeted mass spectrometry (MS)-based metabolomics

Kits are supplied with internal standard applied to each well of the 96-well extraction plate. Ten microliters of each blank, calibration standard, Biocrates QC, Global Reference QC, was added to the appropriate wells. Thirty microliters of each sample and sample pool quality control was added to the appropriate well. The plate was then dried under a gentle stream of nitrogen for 30 minutes. The samples were derivatized with phenyl isothiocyanate then eluted with 5 mM ammonium acetate in methanol. Samples were diluted with either 80:20 water:methanol for the ultra performance liquid chromatography (UPLC) analysis (1:1) or running solvent (a proprietary mixture provided by Biocrates) for flow injection analysis (20:1). Ultra high performance liquid chromatography separation was performed using an Exion AD liquid chromatograph (Sciex, Framingham, MA) with a Waters Acquity 2.1 mm × 50 mm 1.7 µm BEH C18 column fitted with a Waters Acquity BEH C18 1.7 µm Vanguard guard column with a stainless steel pre-column mixer (Biocrates AG, Innsbruck, Austria). Analytes were separated using a gradient from 0.2% formic acid in water to 0.2% formic acid in acetonitrile. Total UPLC analysis time was approximately 6 minutes per sample. Using two injections, one in Electrospray Ionization Positive mode (ESI^+^) and one in negative mode (ESI^−^), samples for UPLC analysis were introduced directly into a QTRAP 6500+ (Sciex) operating in the multiple reaction monitoring (MRM) mode. MRM transitions (compound-specific precursor to product ion transitions) for each analyte and internal standard were collected over the appropriate retention time. The UPLC-MS/MS data were imported into Skyline daily (version 21.1.1.9.335, https://skyline.ms/) for peak integration, calibration, and concentration calculations (62). Export from Skyline was processed using a custom visual basic macro for importing into MetIDQ (Biocrates AG). The flow injection analysis-tandem mass spectrometry (FIA-MS/MS) samples were analyzed in two separate FIA-MS/MS methods with total analysis time of approximately 3.8 minutes per injection using electrospray ionization in ESI^+^ on a Xevo TQ-S triple quadrupole mass spectrometer (Waters). Sample introduction was performed with an Acquity UPLC (Waters). Compound-specific precursor to product ion transitions for each analyte and internal standard were collected. The FIA-MS/MS data for acylcarnitines, monosaccharides (hexose), diglycerides, triglycerides, lysophosphatidylcholines, phosphatidylcholines, sphingomyelins, ceramides, and cholesteryl esters were analyzed using Biocrates MetIDQ software.

### Statistical analyses

A mixed-effect linear model was employed to assess the impact of infection over time on metabolites and metabolism indicators. From this model, effect sizes (fold changes) and adjusted *P*-values were calculated for each metabolite and metabolism indicator across contrasting timepoints. Correlation analysis was performed to assess the relationship between metabolic features and viral load.

## Data Availability

Mass spectrometry data are found in Table S1 in the supplemental material. Further data requests can be made to the Division of Innate and Comparative Immunology (DICI) Data Management team at DICI-data@duke.edu.

## SUPPLEMENTAL MATERIAL

Supplemental material jvi.01634-24-s0001.docx Figures S1 and S2; legend for Table S1.

Table S1 jvi.01634-24-s0002.xls Raw data values of metabolic analytes.

ASM does not own the copyrights to Supplemental Material that may be linked to, or accessed through, an article. The authors have granted ASM a non-exclusive, world-wide license to publish the Supplemental Material files. Please contact the corresponding author directly for reuse.
