# Supplementary material for: Longitudinal analysis of rhesus macaque metabolome during acute SIV infection reveals disruption in broad metabolite classes
Source: J Virol. 2025 Feb 6;99(3):e01634-24. doi: 10.1128/jvi.01634-24 (PMC11915796; doi:10.1128/jvi.01634-24)
Supplement: Supplemental material — Figures S1 and S2; legend for Table S1. [file jvi.01634-24-s0001.docx]

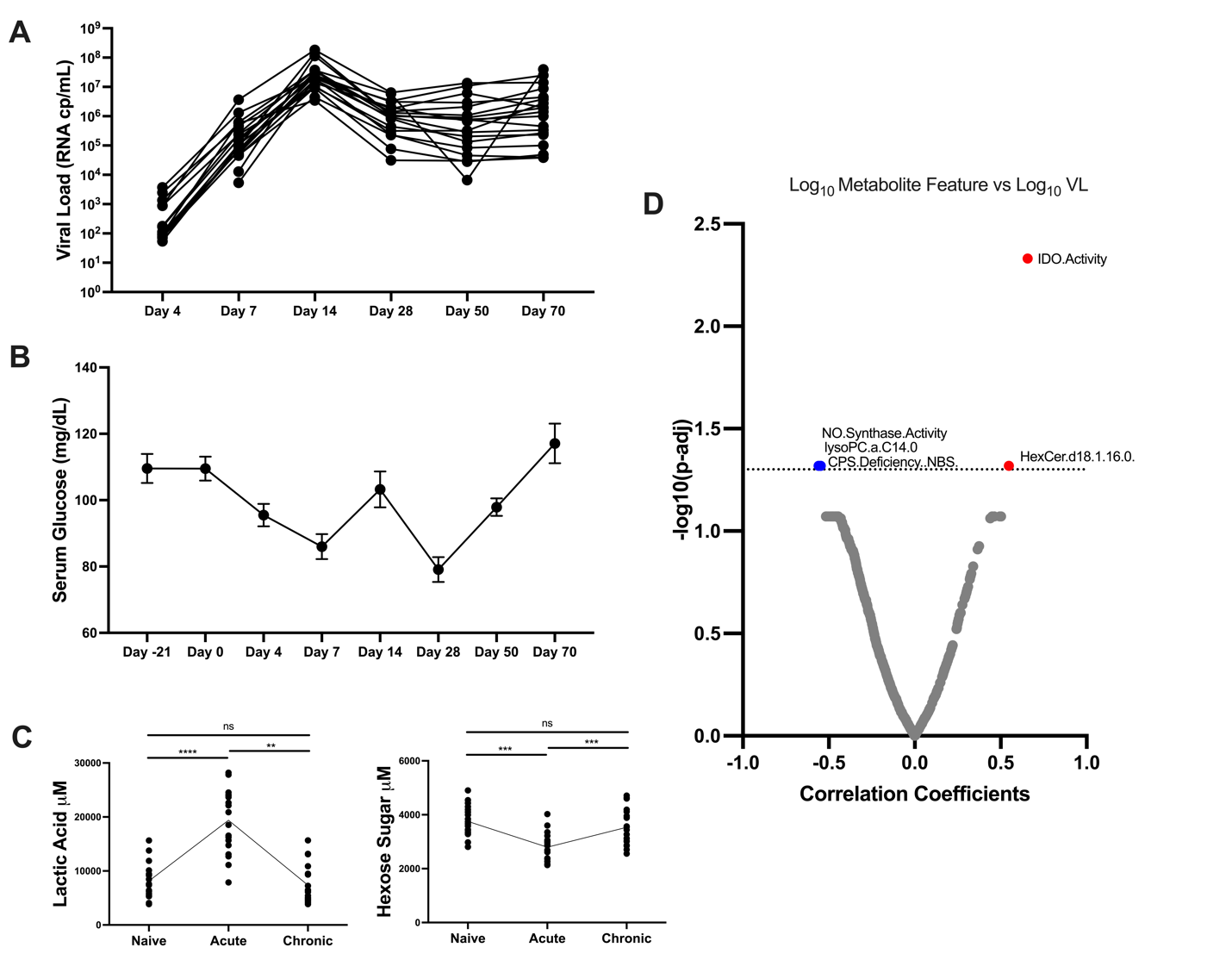


**Figure S1. Viral loads and serum chemistry.** (**A**) VL (RNA cp/mL) in periphery over time. (**B**) Mean serum glucose levels over time. Bars show SEM. (**C**) Longitudinal concentrations of lactic acid and hexose sugar. *p*-values were calculated by paired *t*-test with Benjamini-Hochberg adjustment. (**D**) Correlation of metabolites and VL using naïve, acute, and chronic timepoints, displayed as a volcano plot (Purple = downregulated metabolite, Gray = no significance, Orange = upregulated metabolite; dotted line at *p* = -log_10_(0.05)). Pearson correlation was conducted with Benjamini-Hochberg adjustment for multiple comparisons.

* *p* < 0.05; ** *p* < 0.01; *** *p* < 0.001; **** *p* < 0.0001


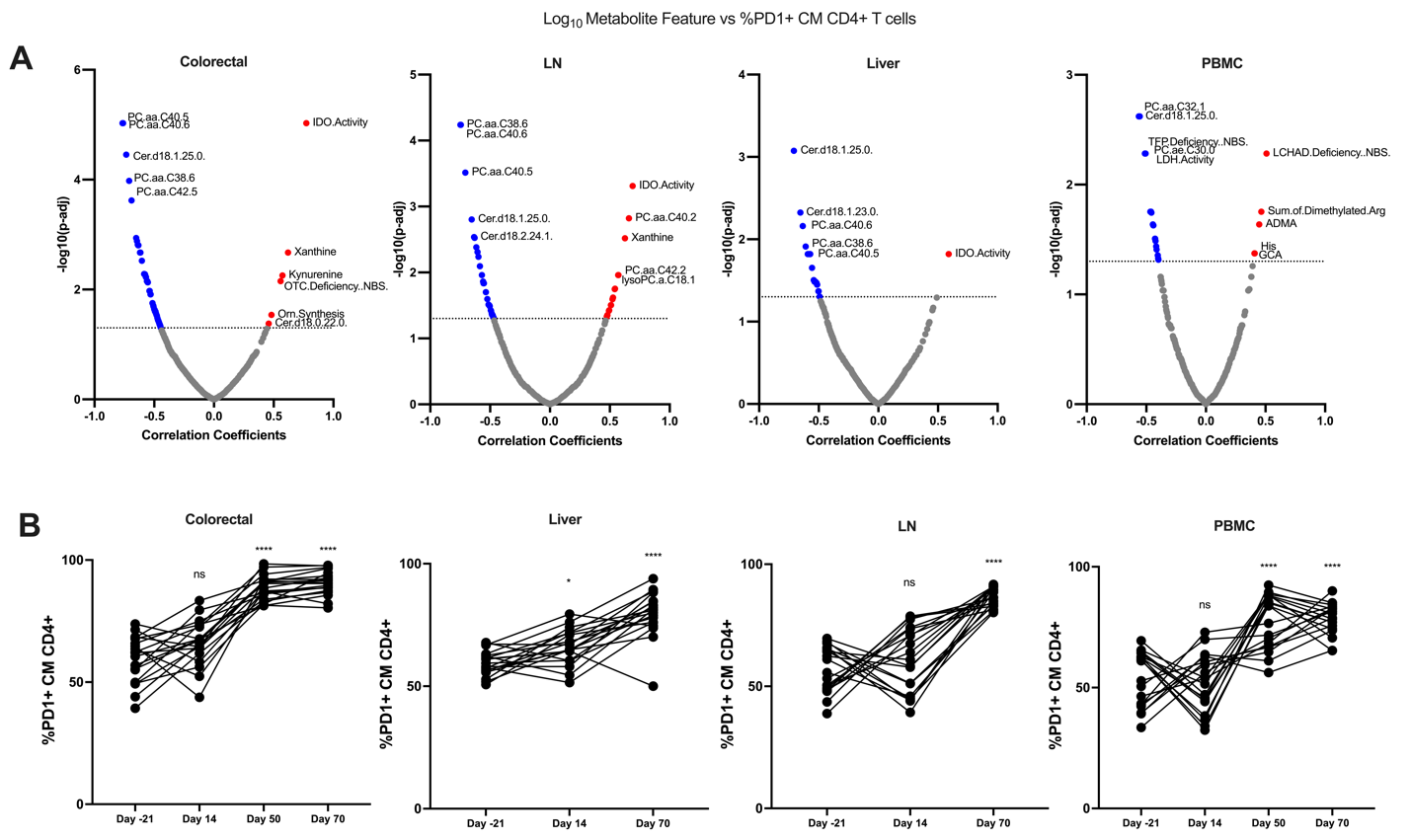


**Figure S2. T cell PD1 expression increases with time and correlates with IDO activity.** (**A**) Correlation of plasma metabolites and frequency of PD1+ cells among CD4+ central memory T cells in colorectal, lymph node, and liver biopsies and in PBMC. (**B**) PD1+ frequency of CD4+ central memory T cells in colorectal, lymph node, and liver biopsies and in PBMC over time. One-way ANOVA was conducted with multiple comparisons against day -21. For volcano plots correlating metabolites with cell populations comparisons are conducted using data from naïve and chronic timepoints. Blue = negatively correlated metabolite, Gray = no significance, Red = positively correlated metabolite; dotted line at *p* = -log_10_(0.05). Pearson correlation was conducted with Benjamini-Hochberg adjustment for multiple comparisons.

* *p* < 0.05; ** *p* < 0.01; *** *p* < 0.001; **** *p* < 0.0001

**Table S1. Raw data values of metabolic analytes.** Metabolic profiling was performed using the MxP^®^ Quant 500 Kit (Biocrates Innsbruck, Austria) in strict accordance with the provided detailed protocol. This specialized kit enabled the assessment of 508 soluble metabolites across various classes by mass spectrometry.
